# A Genetic Screen for *Saccharomyces cerevisiae* Mutants That Fail to Enter Quiescence

**DOI:** 10.1534/g3.115.019091

**Published:** 2015-06-10

**Authors:** Lihong Li, Shawna Miles, Linda L. Breeden

**Affiliations:** Basic Sciences Division, Fred Hutchinson Cancer Research Center, Seattle, Washington 98109

**Keywords:** quiescence, stress tolerance, cell wall, Msn4, Ecm33

## Abstract

Budding yeast begin the transition to quiescence by prolonging G1 and accumulating limited nutrients. They undergo asymmetric cell divisions, slow cellular expansion, acquire significant stress tolerance and construct elaborate cell walls. These morphologic changes give rise to quiescent (Q) cells, which can be distinguished from three other cell types in a stationary phase culture by flow cytometry. We have used flow cytometry to screen for genes that are required to obtain the quiescent cell fraction. We find that cell wall integrity is critical and these genes may help define quiescence-specific features of the cell wall. Genes required to evade the host innate immune response are common. These may be new targets for antifungal drugs. Acquired thermotolerance is also a common property, and we show that the stress-response transcription factors Msn2 and Msn4 promote quiescence. Many other pathways also contribute, including a subset of genes involved in autophagy, ubiquitin-mediated proteolysis, DNA replication, bud site selection, and cytokinesis.

In rich glucose media, yeast cells begin the transition to quiescence by extending the G1 phase of the cell cycle. By the time they cease division, the cells are filled with glucose in the form of trehalose and glycogen (Lillie and Pringle 1980; Li *et al.* 2013). In this way, cells anticipate nutrient limitation and respond by reducing proliferation and stockpiling the critical nutrient. Quiescent (Q) cells comprise a subset of the cells in a stationary phase culture. They can be purified away from the nonquiescent (nonQ) population by density gradient sedimentation. These Q cells are uniformly arrested in G1, highly thermotolerant, and long lived (Allen *et al.* 2006; Li *et al.* 2009, 2013). It has been suggested that the density and the protective properties of quiescent (Q) cells are a result of carbohydrate accumulation (Shi *et al.* 2010). However, introduction of the *SSD1* gene to W303 almost doubles Q cell yield (Li *et al.* 2009) and increases the thermotolerance, longevity and recovery kinetics of Q cells without affecting the levels of storage carbohydrates (Li *et al.* 2013). Hence, carbohydrate accumulation is not solely responsible for these Q cell properties.

When the glucose is exhausted from the medium, W303 cells undergo one more division, which is highly asymmetric and there is also a slowing of physical growth. This results in a dramatic change in modal cell size from 40 to 12 femtoliters (Li *et al.* 2013). These daughter cells preferentially inherit highly functional mitochondria (Lai *et al.* 2002; McFaline-Figueroa *et al.* 2011; Li *et al.* 2013) and undamaged proteins (Aguilaniu *et al.* 2003; Hill *et al.* 2014) and they are the predominant cell type in the Q-cell fraction (Li *et al.* 2013). There is also a gradual accumulation of cells that resist the penetration of the DNA interchelating dye Sytox Green, which results in the appearance of a discrete peak of reduced DNA fluorescence that is characteristic of Q cells.

Just as in log phase cells, the Cln3 cyclin must be down-regulated to achieve G1 arrest. The replication stress checkpoint is active during this interval, and it becomes essential for G1 arrest and viability if Cln3 is overproduced (Miles *et al.* 2013). The transcription repressor Xbp1 is induced after the glucose is exhausted from the medium (referred to as the diauxic shift, or DS) (Mai and Breeden 1997, 2000), and it represses *CLN3* and hundreds of other transcripts after the DS (Miles *et al.* 2013). In the absence of Xbp1, cells undergo additional cell divisions. The resulting dense Q cells are very small and both their longevity and their recovery are compromised.

The unique program of G1 arrest, asymmetric cell division, chromatin reprogramming, and cell wall fortification that takes place as cells transition to quiescence leads to the production of four distinct cell types that can be distinguished by flow cytometry (Li *et al.* 2013). Using fluorescence-activated cell sorting, we showed that one of these cell types (R3) predominates in the Q-cell fraction and hence can be used as a marker for quiescence. We have explored the timing of the log to Q transition by using flow cytometry, and we have used a high-throughput flow cytometry screen of the deletion library of nonessential genes (Tong *et al.* 2001) to identify mutants that fail to produce R3 cells. This screen serves as a starting point for the genetic dissection of the transition to quiescence in budding yeast.

## Materials and Methods

### Strains and growth conditions

We have used BY6500, a *MATa* haploid, prototrophic version of W303 (Li *et al.* 2009) to characterize the transition to quiescence. BY6641, the *SSD1* derivative of BY6500 was also used in Figure 2C. Cells were grown in YEPD medium and samples were taken for flow cytometry as previously reported (Li *et al.* 2013). Q cells were harvested from 7-d cultures and purified by density gradient sedimentation (Allen *et al.* 2006). The yeast deletion library (Tong *et al.* 2001) was grown in rich media (YEPD) with 2% glucose and 100 μg/mL of G418.

### Flow cytometry screen

The yeast deletion library array was first reprinted from the stock copy onto a single-well OmniTray (242811; Nalge Nunc International) by the use of a Beckman biomek2000 robot (serial number 432616). The manual and the robotic arraying procedure have been described (Tong *et al.* 2001). Cells were cultivated on rich medium (YEPD) plates at 30° overnight. The array was then transferred into 96-well microtiter plates (Costar 3598, Fisher) containing 100 μL of liquid YEPD with 100 μg/mL G418 and incubated at 30° for 24 hr. The growth of the cells was stopped and cells were fixed overnight at 4° by adding 200 μL of ethanol. These cells were then pelleted and washed once with H_2_O. Cells were resuspended in 80 μL of 0.2 mg/mL RNaseA in 50 mM Tris-HCl, pH 7.5, and incubated at 37° for 4 hr. Then, 80 μL of Proteinase K in 50 mM Tris-HCl pH 7.5 was added to the cultures at a final concentration of 2 mg/mL, and cultures were incubated at 50° for 1 hr, then 40 μL of 50 mM Tris-HCl with SYTOX Green nucleic acid stain (Invitrogen, Carlsbad, CA) was added to the cultures at final concentration of 1.0 μM. DNA content analysis was performed in the 96-well format using a BD FACS Canto II-2 flow cytometer and Flowjo software (BD Biosciences).

After an initial screening of the deletion array by flow cytometry, deletion mutants that showed different distributions of cell types were retested. Mutants were grown in 5 mL of YEPD with G418 overnight at 30°, cell cultures were then diluted to OD 0.02 and grown for 24 hr growth at 30°, 200 rpm in culture tubes. A total of 50 μL of each culture was fixed with 70% of ethanol overnight. Cells were washed with water and incubated with 0.2 mg/mL RNase A in 50 mM Tris·HCl, pH 8, for 4 hr at 37°. The cells were spun down and incubated with 2 mg/mL proteinase K in 50 mM Tris·HCl, pH 7.5, at 50° for 1 hr. The cells were then washed with 50 mM Tris·HCl, pH 7.5, sonicated 10 sec at a low setting, then resuspended in 50 mM Tris·HCl, pH 7.5, with 1 μM Sytox-Green. Stained cells were analyzed on a FACScan cytometer in 96 well trays or in standard FACS tubes.

## Results and Discussion

### Stationary phase cells differentiate into four distinct cell types that can be followed by flow cytometry

**Figure 1:**
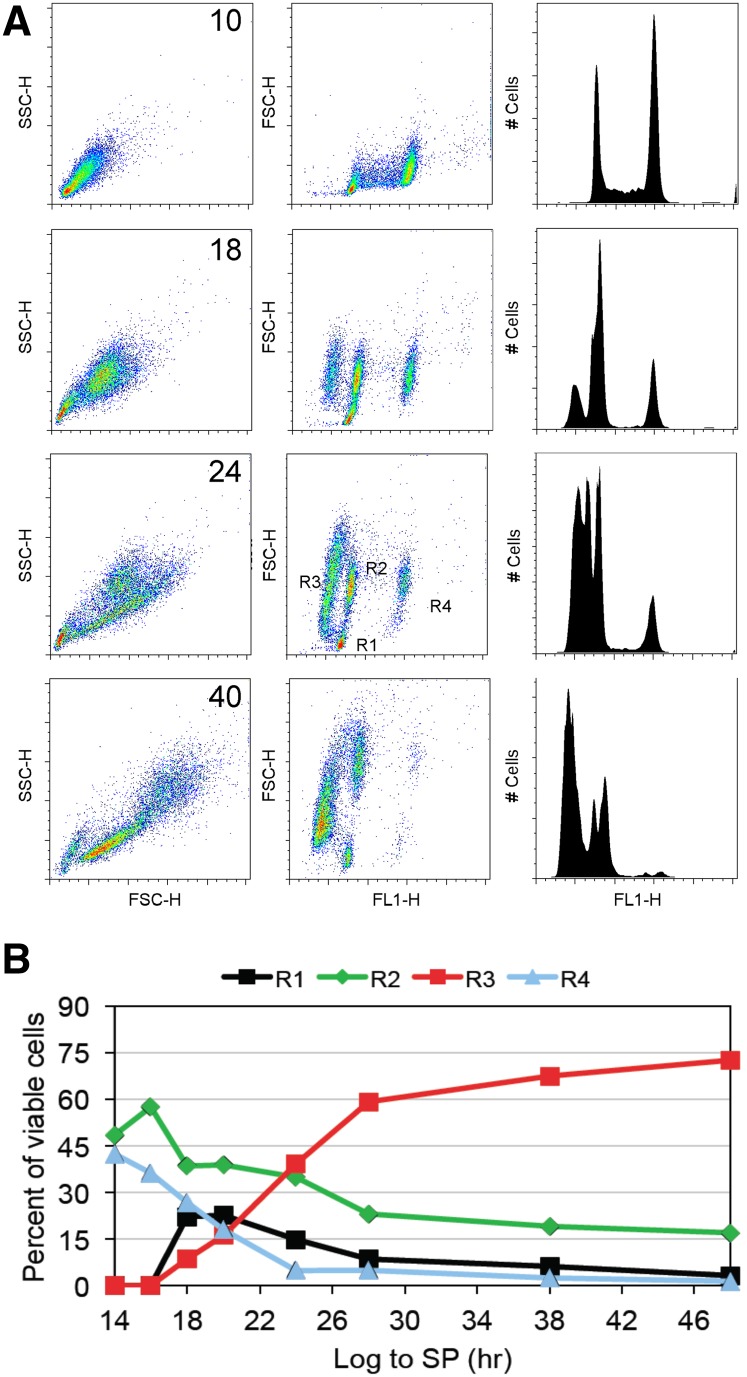
Stationary phase cultures differentiate into four distinct cell types. (A) A combination of forward and side light scattering (FSC-H and SSC-H) and DNA fluorescence (FLH-1) enables the tracking and quantification (B) of cell types as they transit from log phase to stationary phase. R2 and R4 cells have the properties of log phase cells; these are primarily mother cells in G1 or G2/M. R1 cells are the small daughter cells that are the product of asymmetrical cell division. These are the first unique cells to be produced and are the likely precursors to the R3 cells, which are also daughter cells. R3 cells scatter more light but have reduced DNA fluorescence.

**Figure 2:**
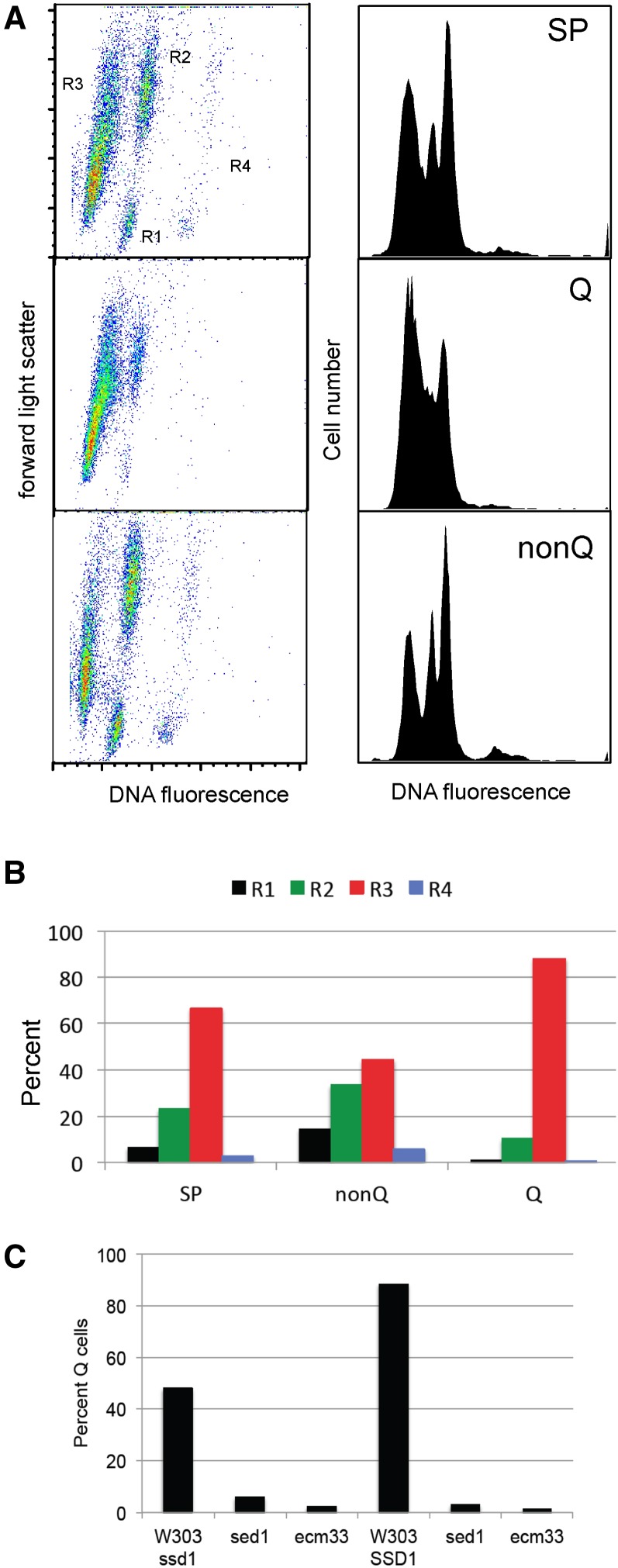
Purified Q cells are primarily R3 cells. (A) Stationary phase (SP) cultures were fractionated into Q and nonQ fractions by density gradient sedimentation and the cell types within these fractions were assayed and quantified (B) by flow cytometry. (C) Cell wall proteins Sed1 and Ecm33 are required for Q-cell formation.

After 10 hr of growth, at an optical density (OD_600_) of about 1, wild-type yeast cells are in the logarithmic phase of growth. These growing cells display uniform light scattering, despite the fact that they are in all phases of the cell cycle (Figure 1A, upper left panel). Forward light scattering combined with DNA fluorescence (middle panel) or fluorescence emission alone (right panel) shows the familiar pattern with two discrete peaks of 1N and 2N DNA content that identify cells that are in G1 and G2/M, and a spread of cells between the peaks that identify cells that are in S phase. After 16 hr, we begin to see more heterogeneous light scattering and a new discrete peak of reduced fluorescence (Figure 1A). Both get more prominent with time. After 24 hr, the 1N cells have split into very discrete populations and there are four different cell types (R1-R4). On the basis of fluorescence activated cell sorting (Li *et al.* 2013), we know that the R1 cells are very small daughter cells. They are all in G1 and have a slightly reduced DNA fluorescence intensity compared to the R2 cells. The R2 and R4 cells form one group by light scattering, but they can be split into two groups based on DNA fluorescence. These include all of the old mother cells that are in G1 (R2) and G2/M (R4). R3 contains daughter cells and some young mothers. They are clearly larger than the R1 daughters and they display less DNA fluorescence. Purified Q cells are primarily R3 cells (Figure 2).

We have quantified the differentiation of these classes over 50 hr as cells transit from logarithmic growth, through the diauxic shift and to the Q state (Figure 1B). Plots for the entire time course are provided in Supporting Information, Figure S1. Under the conditions we employ, the diauxic shift occurs at 14 hr. At 18 hr, the small R1 daughters are at their peak. This population appears before the R3 cells appear and drops over time as the R3 cell population increases. This is consistent with the small R1 daughter cells being precursors of the larger R3 daughters. The disappearance of the R4 cells shows that G1 arrest is nearly complete by 24 hr. After 28 hr, when 95% of the cells are in G1, the R3 population becomes predominant. This population continues to increase and comprises 75% of the culture by 50 hr. At 50 hr, cell division has ceased at about 4 × 10^8^ cells per milliliters and an OD_600_ of approximately 20 (Miles *et al.* 2013).

After 7 d of growth, all four cell types can still be detected in stationary phase cultures (Figure 2), and we find that the fraction of each type varies in both wild and laboratory yeast strains (data not shown). With prototrophic W303 cells, the purified Q cells are all in G1, 90% are R3 cells and 10% are small R2 cells, which are young mother cells (Li *et al.* 2013). The nonquiescent (nonQ) population that remains at the top of the gradient is a mixture of all four cell types. The small population of R1 cells that remains after seven days in culture all fractionate as nonQ cells. They have not achieved the density shift characteristic of Q cells. The R2 cells in the nonQ population scatter more light than those found in the Q fraction, which is consistent with them being the older, larger mother cells. The nonQ population also includes a significant fraction of R3 cells. These R3 cells are less heterogeneous and scatter less light than Q cells, suggesting that their cell walls are less elaborate than those of the Q cells. The fact that almost half of the nonQ cells are in the R3 population suggests that the events that reduce DNA fluorescence are independent of the events that confer high density to the cells. Nevertheless 90% of the Q cells are R3 cells, so it may be a necessary but not sufficient step in the process of Q cell differentiation.

We have previously shown that elimination of Sed1, one of the major cell wall proteins in stationary phase cells (Shimoi *et al.* 1998) eliminates the R3 population (Li *et al.* 2013). To see whether cell wall defects also influence Q cell production, we deleted *SED1* and another cell wall protein gene *ECM3* from W303 prototrophs carrying either *ssd1* or *SSD1*, which also influence Q cell formation (Li *et al.* 2009). These cells were grown for seven days and fractionated by density gradient sedimentation. Neither mutant produced R3 cells (Li *et al.* 2013) and data not shown), and neither mutant produced a significant number of Q cells in either background (Figure 2C). This indicates that the production of R3 cells, whatever it entails, is important for the formation of dense Q cells. As such, the absence of R3 cells can be used to screen for mutants that fail to make this transition.

### A high-throughput screen for mutants that fail to enter quiescence

After 18 hr of growth, about 20% of the cells are tiny R1 cells, and about 10% are R3 cells (Figure 1). Six hours later, most of the cells have G1 DNA content and the R1 and R3 cell abundance shifts to where there are almost three times more R3 cells than R1 cells. On the basis of this analysis, we chose the 24-hr time point to screen the yeast deletion library for mutants that fail to produce R3 cells. Deletion mutants were grown in 96 well plates for 24 hr in rich medium and then analyzed by high-throughput flow cytometry.

Our data indicate that R3-deficient (Rtd−) mutants will include mutants that disrupt or disorganize the cell wall. These cell wall defects may increase cell permeability. They may also induce a stress response (Levin 2011; Ragni *et al.* 2011) and change the ionic environment of the DNA, among other things. We have assayed the DNA fluorescence of cells immediately after dye addition and then again after 4 d of incubation with dye and found no increase in DNA fluorescence at the later time point (Figure S2A). This indicates that only the dye that enters the cells immediately can bind to the DNA, and even prolonged incubation doesn’t enable more dye to enter and/or bind. We have also experimented with extra RNAse and protease treatments of cells before adding dye and we were unable to increase DNA fluorescence of Q cells (Figure S2B). This finding suggests that cell permeability is not the only obstacle to dye binding. Other processes that reduce the interchelation of dyes into Q DNA (*e.g.*, compaction, sequestration or modification of the DNA) could also be in play.

**Figure 3:**
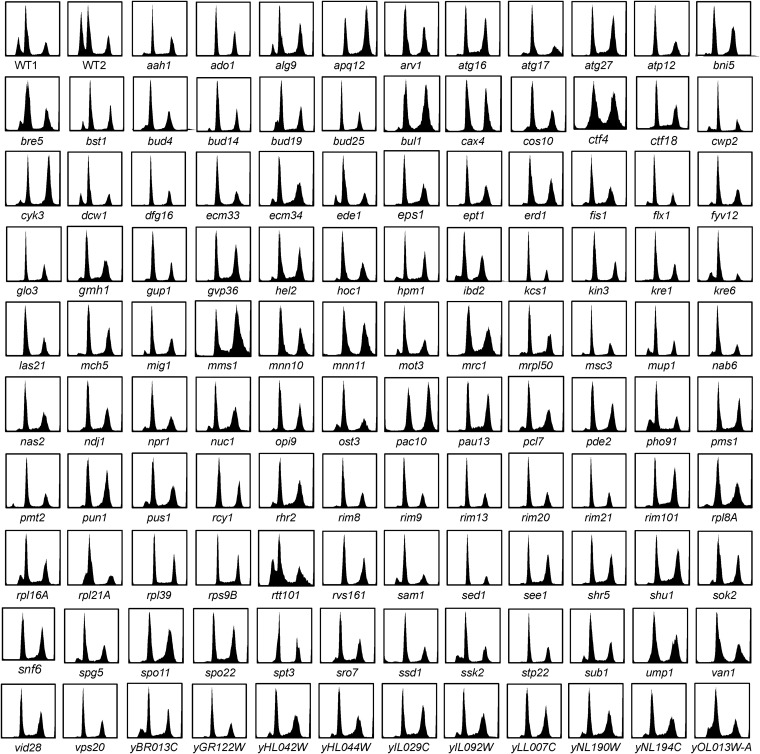
DNA fluorescence histograms of R3-deficient (Rtd−) mutants after 24 hr of growth in rich glucose medium. The first two histograms (WT1 and WT2) reflect the extent of variation we observe with wild-type cells.

If there is a unique conformation of R3 DNA, we might identify mutants that influence that process. We also expect to find mutants that fail in the upstream recognition and/or signaling events that initiate the transition to quiescence. There may be other mutants that are unable to cope with the stresses that arise during this metabolic transition. Such defects could lead to slow growth, death, or a checkpoint arrest, any of which could delay, obscure, or prevent the processes that lead to R3 cell formation. With this in mind, we screened the deletion library of nonessential genes (Tong *et al.* 2001) for Rtd− mutants. Here, we report the identification of 118 mutants that produce little or no R3 cells (Table 1). Parentheses indicate Rtd− mutants that are adjacent to another Rtd− mutant. In these cases, it is possible that the Rtd− phenotype shared by adjacent genes is conferred by loss of only one of the genes, but the disruption of the adjacent gene interferes with transcription of its neighbor (Alvaro *et al.* 2007). For example, there is a cluster of four Rtd− mutants (YHL042W to YHL046C) that are unlikely to all be required to form R3 cells (Table 1). In this case, it may be that disruption of one or more of these loci is interfering with the transcription of one gene in the cluster that is conferring the Rtd− phenotype. This has been referred to as neighboring gene effect (Ben-Shitrit *et al.* 2012). Representative DNA fluorescence histograms of all 118 Rtd− mutants are shown in Figure 3.

**Table 1:** R3-deficient (Rtd−) deletion mutants

ORF ID	Gene Name	Trait	Description
Cell wall organization and biogenesis			
YKL046C	*DCW1*	2,4	Putative mannosidase, GPI-anchored membrane protein required for cell wall biosynthesis in bud formation
YJR075W	*HOC1*	1,2,5	Alpha-1,6-mannosyltransferase; involved in cell wall mannan biosynthesis; in Golgi complex of Anp1p, Mnn9, 10, and 11
YDR245W	*MNN10*	2,5	Subunit of a Golgi mannosyltransferase complex also containing Anp1p, Mnn9p, Mnn11p, and Hoc1p
YJL183W	*MNN11*	2,3,5	Subunit of a Golgi mannosyltransferase complex that also contains Anp1p, Mnn9p, Mnn10p, and Hoc1p
YKL096W-A	*CWP2*	2	Covalently linked cell wall mannoprotein, major constituent of the cell wall; plays a role in stabilizing the cell wall
YNL322C	*KRE1*	2	Cell wall glycoprotein involved in beta-glucan assembly; serves as a K1 killer toxin membrane receptor
YPR159W	*KRE6*	2,5	Required for beta-1,6 glucan biosynthesis, localizes to ER, plasma membrane, sites of polarized growth, and secretory vesicles
YBR078W	*ECM33*	2,5	GPI-anchored protein of unknown function, has a possible role in apical bud growth
YNL190W	*YNL190W*		Cell wall protein; proposed role as a hydrophilin induced by osmotic stress; contains a putative GPI-attachment site
YDR077W	*SED1*	2	Major stress-induced structural cell wall glycoprotein in stationary-phase cell
YDR293C	*SSD1*	1,3,4,5	RNA-binding protein with a role in maintenance of cellular integrity, interacts with components of the TOR pathway
YNL294C	*RIM21*	4	Component of the RIM101 pathway, has a role in cell wall construction and alkaline pH response
YML117W	*NAB6*	1	Putative RNA-binding protein that associates with mRNAs encoding cell wall proteins in high-throughput studies
Protein lipidation			
YLR242C	*ARV1*	2,6	Protein functioning in transport of GPI intermediates into ER lumen
YGL084C	*GUP1*	2,5	Plasma membrane protein involved in remodeling GPI anchors; proposed to be involved in glycerol transport
YJL062W	*LAS21*	1	Integral plasma membrane protein involved in the synthesis of the GPI core structure
YOL110W	*SHR5*	1	Subunit of a palmitoyltransferase, palmitoylation is required for Ras2p membrane localization
Glycoprotein biosynthesis			
YDR414C	*ERD1*	4	Predicted membrane protein required for the retention of lumenal endoplasmic reticulum proteins
YJR075W	*HOC1*	1,2,5	Alpha-1,6-mannosyltransferase; subunit of a Golgi complex of Anp1p, Mnn9p, Mnn11p, and Mnn10p
YDR245W	*MNN10*	2,5	Subunit of a Golgi mannosyltransferase complex also containing Anp1p, Mnn9p, Mnn11p, and Hoc1p
YJL183W	*MNN11*	2,3,5	Subunit of a Golgi mannosyltransferase complex that also contains Anp1p, Mnn9p, Mnn10p, and Hoc1p
YML115C	*VAN1*	1,2	Mannan polymerase I subunit involved in the first steps of mannan synthesis
YAL023C	*PMT2*	2	Protein *O*-mannosyltransferase, transfers mannose to protein Ser/Thr residues
YNL219C	*ALG9*	1,2,6	Mannosyltransferase, involved in N-linked glycosylation
YGR036C	*CAX4*	2	Dolichyl pyrophosphate (Dol-P-P) phosphatase in the ER, required for protein N-glycosylation
YOR085W	*OST3*	1,2	Oligosaccharyltransferase complex subunit of the ER lumen; important for N-glycosylation of a subset of proteins
Vesicle-mediated transport			
YBL047C	*EDE1*	5	Key endocytic protein involved in a network of interactions with other endocytic proteins
YNL106C	*INP52*		Polyphosphatidylinositol phosphatase, dephosphorylates a number of PIs to PI; involved in endocytosis
YJL204C	*RCY1*	5	F-box protein involved in recycling plasma membrane proteins internalized by endocytosis
YCR009C	*RVS161*	3,5	BAR domain lipid raft protein; regulates polarization of the actin cytoskeleton, endocytosis, cell polarity fusion
YIL041W	*(GVP36)*	4	BAR domain−containing protein in Golgi; required for adaptation to varying nutrient concentrations, endocytosis and actin polarization
YFL025C	*BST1*		GPI inositol deacylase of the ER that negatively regulates COPII vesicle formation
YNR051C	*BRE5*	5	Ubiquitin protease cofactor, forms deubiquitination complex with Ubp3p
YIL064W	*SEE1*		Probable lysine methyltransferase involved in the dimethylation of eEF1A (Tef1p/Tef2p); role in vesicular transport
YNR013C	*PHO91*		Vacuolar phosphate transporter, exports phosphate from vacuole to cytosol, overexpression results in vigorous growth
YMR077C	*(VPS20)*	4,5	Subunit of ESCRTIII, the endosomal sorting complex required for transport of transmembrane proteins to the vacuolar lumen
YPR032W	*SRO7*	4	Effector of Rab GTPase Sec4p; involved in exocytosis and fusion of post-Golgi vesicles with plasma membrane;
YER122C	*GLO3*	5	ADP-ribosylation factor GTPase activating protein, involved in ER−Golgi transport
YNR075W	*COS10*		Protein of unknown function, member of the DUP380 subfamily of conserved, often subtelomerically encoded proteins
YGL045W	*RIM8*		Involved in proteolytic activation of Rim101p in response to alkaline pH; interacts with ESCRT-1 subunits Stp22p and Vps28p
YNL183C	*NPR1*	4	Kinase that stabilizes plasma membrane amino acid transporters by antagonizing their ubiquitin-mediated degradation
YKR030W	*GMH1*		Golgi membrane protein, possible role in either cell wall synthesis or protein-vacuolar targeting
YDR017C	*KCS1*	5	Inositol hexa- and heptakisphosphate (IP7) kinase, vacuole biogenesis, and endocytosis
YIL005W	*EPS1*		ER chaperone protein, involved in retention of resident ER proteins and recognizing proteins targeted for degradation (ERAD)
Sites of polarized growth			
YJR092W	*BUD4*		Protein involved in bud-site selection and required for axial budding pattern
YAR014C	*BUD14*		Protein involved in bud-site selection, Bud14p-Glc7p complex is a cortical regulator of dynein; inhibitor of the actin assembly factor Bnr1p
YJL188C	*(BUD19)*		Putative protein involved in bud-site selection
YER014C-A	*BUD25*		Protein involved in bud-site selection; diploid mutants display a random budding pattern instead of the wild-type bipolar pattern
YNL166C	*BNI5*	4	Protein involved in organization of septins at the mother-bud neck, may interact directly with the Cdc11p septin
YLR414C	*PUN1*	3,5,6	Putative protein of unknown function; localizes to bud and cytoplasm
YDL117W	*CYK3*	2	SH3-domain protein located in the mother-bud neck and the cytokinetic actin ring
YNR031C	*SSK2*	1,6	Mitogen-activated protein kinase kinase kinase of the HOG1 mitogen-activated signaling pathway
YDR293C	*SSD1*	1,3,4,5	RNA-binding protein with a role in maintenance of cellular integrity, interacts with components of the TOR pathway
YPR159W	*KRE6*	2,5	Required for beta-1,6 glucan biosynthesis, localizes to ER, plasma membrane, sites of polarized growth and secretory vesicles
YBL047C	*EDE1*	5	Key endocytic protein involved in a network of interactions with other endocytic proteins
YJL204C	*RCY1*	5	F-box protein involved in recycling plasma membrane proteins internalized by endocytosis; localized to sites of polarized growth
YCR009C	*RVS161*	3	Lipid raft protein; interacts with Rvs167p, regulates polarization of the actin cytoskeleton, endocytosis, cell polarity and fusion
Rim101 function			
YHL027W	*RIM101*	4	Transcriptional repressor involved in response to pH and in cell wall construction; required for haploid invasive growth and sporulation
YMR154C	*RIM13*	4,6	Calpain-like cysteine protease involved in proteolytic activation of Rim101p in response to alkaline pH
YOR275C	*RIM20*	4,5	Protein involved in proteolytic activation of Rim101p in response to alkaline pH; PalA/AIP1/Alix family member
YNL294C	*RIM21*	4	Component of the RIM101 pathway, has a role in cell wall construction and alkaline pH response
YGL045W	*RIM8*	4	Protein involved in proteolytic activation of Rim101p in response to alkaline pH; interacts with ESCRT-1
YMR063W	*RIM9*	4,5,6	Protein of unknown function, involved in the proteolytic activation of Rim101p in response to alkaline pH
YOR030W	*DFG16*	4,6	Probable multiple transmembrane protein; involved in diploid invasive and pseudohyphal growth upon nitrogen starvation
YGR122W	*YGR122W*	4	Probable ortholog of *A. nidulans* PalC, which is involved in pH regulation and binds to the ESCRT-III complex
Transcription factors			
YHL027W	*RIM101*	4	Repressor involved in response to pH and in cell wall construction; required for haploid invasive growth and sporulation
YMR016C	*SOK2*	5	Nuclear protein that plays a regulatory role in the cyclic AMP (cAMP)-dependent protein kinase (PKA) signal transduction pathway
YGL035C	*MIG1*	1,5,6	Transcription factor involved in glucose repression; sequence specific DNA binding protein containing two Cys2His2 zinc finger motifs
YHL025W	*SNF6*	5,6	Subunit of the SWI/SNF chromatin remodeling complex involved in transcriptional regulation
YMR070W	*MOT3*	4	Nuclear transcription factor with two Cys2-His2 zinc fingers; involved in repression of a subset of hypoxic genes by Rox1p
YMR039C	*SUB1*	1,6	Transcriptional coactivator, facilitates elongation through factors that modify RNAP II; role in peroxide resistance involving Rad2p
YDR392W	*SPT3*	3,5,6	SAGA subunit, regulates RNA Polymerase II-dependent genes
Ubiquitin-dependent protein catabolism			
YMR275C	*BUL1*	1	Ubiquitin-binding component of the Rsp5p E3-ubiquitin ligase complex
YPR164W	*MMS1*	1	E3 ubiquitin ligase component involved in replication, regulates Ty1 transposition
YDR266C	*HEL2*		Ring finger E3 ubiquitin ligase, localizes to the cytoplasm, degrades histones
YIL007C	*NAS2*		Proteasome-interacting protein involved in the assembly of the base subcomplex of the 19S proteasomal regulatory particle
YIL017C	*VID28*	1,5	Protein involved in proteasome-dependent catabolite degradation of fructose-1,6-bisphosphatase (FBPase)
YBR173C	*UMP1*		Chaperone required for maturation of 20S proteosome
YMR191W	*SPG5*		Protein required for survival at high temperature during stationary phase
YMR077C	*(VPS20)*	4,5	ESCRTIII subunit, the endosomal sorting complex required for transport of transmembrane proteins to the vacuole
YCL008C	*STP22*	1,5	Component of the ESCRT-I complex, which is involved in ubiquitin-dependent sorting of proteins into the endosome
YMR154C	*RIM13*	4,6	Calpain-like cysteine protease involved in proteolytic activation of Rim101p in response to alkaline pH
YOR275C	*RIM20*	4,5	Protein involved in proteolytic activation of Rim101p in response to alkaline pH; PalA/AIP1/Alix family member
YAL023C	*PMT2*	2	Protein O-mannosyltransferase, transfers mannose residues from dolichyl phosphate-D-mannose to protein Ser/Thr residues
YIL005W	*EPS1*		ER chaperone involved in retention of ER proteins and recognizing proteins targeted for ER-associated degradation
Autophagy			
YLR423C	*ATG17*	1	Scaffold protein responsible for phagophore assembly site organization; stimulates Atg1p kinase activity
YMR159C	*ATG16*	1	Forms Atg12p-Atg5p-Atg16p multimers, required for autophagy
YJL178C	*ATG27*	1	Type I membrane protein involved in autophagy and the cytoplasm-to-vacuole targeting (Cvt) pathway
YNR051C	*BRE5*	5,6	Ubiquitin protease cofactor, forms deubiquitination complex with Ubp3p
DNA replication and repair			
YNL082W	*PMS1*	4	ATP-binding protein required for mismatch repair in mitosis and meiosis; functions as a heterodimer with Mlh1p
YOL104C	*NDJ1*		Meiosis-specific telomere protein, required for chromosomal segregation and telomere-led rapid prophase movement
YLR219W	*MSC3*	6	unknown function, GFP-fusion protein localizes to the cell periphery, meiotic recombination defective
YHL006C	*SHU1*		Rad51p-, Rad54p-dependent pathway for homologous recombination repair and error-free repair of DNA lesions
YHL022C	*SPO11*		Meiosis-specific, initiates meiotic recombination; required for homologous chromosome pairing and synapsis
YIL073C	*SPO22*	4	Meiosis-specific, essential for chromosome synapsis, involved in completion of nuclear divisions during meiosis
YNL194C	*YNL194C*		Integral membrane protein required for sporulation and plasma membrane sphingolipid content
YCL061C	*MRC1*	3	S-phase checkpoint protein required for DNA replication; protects uncapped telomeres
YPR164W	*MMS1*	1	E3 ubiquitin ligase component involved in replication, regulates Ty1 transposition
YPR135W	*CTF4*		Required for sister chromatid cohesion; and may link DNA synthesis to sister chromatid cohesion
YMR078C	*(CTF18)*		Required for sister chromatid cohesion; may function in the DNA damage replication checkpoint
YAR018C	*KIN3*	5	Required for checkpoint arrest in response to DNA damage
Translation			
YJL189W	*(RPL39)*		Protein component of the large (60S) ribosomal subunit; required for ribosome biogenesis
YHL033C	*RPL8A*	1,3	Ribosomal protein L4 of the large (60S) ribosomal subunit, nearly identical to Rpl8Bp
YBR189W	*RPS9B*		Protein component of the small (40S) ribosomal subunit; nearly identical to Rps9Ap
YIL133C	*(RPL16A)*	1	N-terminally acetylated protein component of the large (60S) ribosomal subunit, binds to 5.8 S rRNA
YBR191W	*RPL21A*		Protein component of the large (60S) ribosomal subunit, nearly identical to Rpl21Bp
YPL212C	*PUS1*	5	tRNA:pseudouridine synthase; nuclear protein that appears to be involved in tRNA export; also acts on U2 snRNA
YIL110W	*HPM1*	1,4	AdoMet-dependent methyltransferase involved in a novel 3-methylhistidine modification of ribosomal protein Rpl3p
Mitochondrial function			
YJL208C	*NUC1*	5	Major mitochondrial nuclease, roles in mitochondrial recombination, apoptosis and maintenance of polyploidy
YNR022C	*MRPL50*		Mitochondrial ribosomal large subunit protein, not essential for translation
YJL180C	*ATP12*	5	Conserved protein required for assembly of alpha and beta subunits into the F1 sector of mitochondrial F1F0 ATP synthase
YIL134W	*(FLX1)*		Required for transport of FAD, a synthesis product of riboflavin
YIL065C	*FIS1*	1,4	Protein involved in mitochondrial membrane fission and peroxisome abundance
Other			
YOR360C	*PDE2*	1,3,5	High-affinity cyclic AMP phosphodiesterase, component of the cAMP-dependent protein kinase signaling system
YNL164C	*IBD2*		Component of the BUB2-dependent spindle checkpoint pathway, interacts with Bfa1p and functions upstream of Bub2p and Bfa1p
YIL050W	*PCL7*		Pho85p cyclin of the Pho80p subfamily, regulated by Pho81p; involved in glycogen metabolism
YIL053W	*RHR2*	4	DL-glycerol-3-phosphatase; involved in glycerol biosynthesis
YDR017C	*KCS1*	5	Inositol hexakisphosphate (IP6) and inositol heptakisphosphate (IP7) kinase
YGR078C	*PAC10*	5,6	Part of the heteromeric co-chaperone GimC/prefoldin complex, which promotes efficient protein folding
YNL141W	*AAH1*		Adenine deaminase (adenine aminohydrolase), converts adenine to hypoxanthine; involved in purine salvage
YJR105W	*ADO1*	5	Adenosine kinase, required for the utilization of S-adenosylmethionine (AdoMet)
YLR180W	*SAM1*		S-adenosylmethionine synthetase, catalyzes transfer of the adenosyl group of ATP to the sulfur atom of methionine
YGR055W	*MUP1*		High affinity methionine permease, integral membrane protein with 13 putative membrane-spanning regions
YOR306C	*MCH5*	5	Plasma membrane riboflavin transporter; facilitates the uptake of vitamin B2; required for FAD-dependent processes
YHR123W	*EPT1*		sn-1,2-diacylglycerol ethanolamine- and cholinephosphotranferase; not essential for viability
YIL040W	*(APQ12)*	6	Protein required for nuclear envelope morphology, nuclear pore complex localization, mRNA export from the nucleus
YOR183W	*FYV12*		Protein of unknown function, required for survival upon exposure to K1 killer toxin
YHL042W	*(YHL042W)*		Member of the DUP380 subfamily of conserved, often subtelomerically-encoded proteins
YHL043W	*(ECM34)*		Member of the DUP380 subfamily of conserved, often subtelomerically-encoded proteins
YHL044W	*(YHL044W)*	4	Putative integral membrane protein, member of DUP240 gene family
YHL046C	*(PAU13)*		Member of the seripauperin multigene family encoded mainly in subtelomeric regions
YLL007C	*YLL007C*	5	Putative protein of unknown function; GFP-fusion protein localizes to the cytoplasm
YLR338W	*OPI9*	5	Dubious open reading frame unlikely to encode a protein, partially overlaps the verified ORF VRP1/YLR337C.
YOL013W-A	*YOL013W-A*		Putative protein of unknown function; identified by SAGE
YBR013C	*YBR013C*		Unknown function, haploid mutant exhibits synthetic phenotype with alpha-synuclein
YIL029C	*YIL029C*	4,5	Deletion confers sensitivity to 4-(N-(S-glutathionylacetyl)amino) phenylarsenoxide (GSAO)
YIL092W	*YIL092W*		GFP-fusion protein localizes to the cytoplasm and to the nucleus

Descriptions are derived from Saccharomyces Genome Database. Parentheses indicate genes adjacent to other Rtd− genes. Traits are: 1, desiccation sensitive (Ratnakumar and Tunnacliffe 2006); 2, cell wall protein (Orlean 2012); 3, acquired thermotolerance defective (Mir *et al.* 2009); 4, antimicrobial peptide sensitivity (Lis *et al.* 2013); 5, ethanol sensitive (Avrahami-Moyal *et al.* 2012); and 6, Set3 complex interaction (Kim *et al.* 2012). GPI, glycosylphosphatidylinositol; ER, endoplasmic reticulum; TOR, target of rapamycin; PIs, phosphatidylinositols; ESCRTs, endosomal sorting complexes required for transport; GFP, green fluorescent protein; FAD, flavin adenine dinucleotide.

### Cell wall−related genes required for Q-cell formation

As expected, many of the Rtd− mutants that we identified have other cell wall-associated phenotypes and could be directly or indirectly involved in Q-cell wall formation. In fact, 33 of these 118 gene products are localized to the cell periphery (*P*-value 10^−7^). Other significantly enriched Gene Ontology terms are noted with their associated p-values in parentheses.

The cell wall is the major point of contact and the first line of defense from environmental stress and host immune responses (Levin 2011; Hall and Gow 2013). The cell wall makes up about 20% of the dry weight of a yeast cell and involves at least 180 proteins (Orlean 2012). Many of the structural and enzymatic components of the cell wall are highly conserved, and others show signs of rapid evolution. The cell wall defines the shape, osmo-stability and immunogenicity of yeast cells. Besides its protective role, the cell wall must be readily remodeled to carry out mating, morphogenesis, cell duplication, and growth arrest when internal or external conditions dictate. Finally, the cell wall must be equipped to permit the regulated import of critical nutrients and the export of cell surface proteins, extracellular signaling molecules and toxic byproducts of metabolism. The importance of this dynamic structure for the survival and pathogenicity of fungi has led to many directed efforts to biochemically and genetically define the components of the cell wall (Orlean 2012). Our screen contributes to this effort. We have identified many of the genes already implicated in cell wall biogenesis. In addition, we expect to identify activities unique to forming the quiescent cell wall, which our light scattering data show is significantly different than the cell wall of exponentially growing cells.

Sed1 and Ecm33 are required for the reduced DNA fluorescence of R3 cells, and we identified both *sed1* and *ecm33* in this screen for reduced R3 cells (Table 1). In addition, we found 19 other mutants that encode enzymes that are directly involved in the biosynthesis or remodeling of the cell wall or are nonenzymatic cell wall proteins (Orlean 2012). These genes include enzymes involved in cell wall protein modification, *e.g.*, mannosylation (*P* = 0.008), lipidation, and glycosylation (*P* = 0.0015). We also found 17 mutants involved in vesicle-mediated transport (*P* = 0.26), which may deliver cell wall proteins to the endoplasmic reticulum (*P* = 0.05) or the Golgi (*P* = 0.0002), where these modifications take place (Orlean 2012). Among these are two of the three BAR domain proteins of budding yeast (*GVP36* and *RVS161*). These are lipid raft proteins that play important roles in endocytosis (*P* = 0.047) and actin polarization and are known to be required for viability in stationary phase (Crouzet *et al.* 1991; Querin *et al.* 2008).

### The stress resistance and quiescence pathways show significant overlap

One of the first types of environmental stress that was used to specifically identify cell wall components involved agents that directly interact with the cell surface (*e.g.*, detergents, sonication, calcofluor, and Congo red). An early screen of 600 mutants with three such stresses suggested that up to one-quarter of *S. cerevisiae* genes may indirectly impact the cell wall (de Groot *et al.* 2001). Only nine mutants that we identified were among those tested and found to be sensitive. This suggests that we are primarily picking up a different class of mutants.

A critical function of the cell wall is to confer resistance to common environmental stresses like desiccation and heat. Desiccation tolerance is a known property of stationary phase cells and the majority of cells acquire desiccation tolerance after the diauxic shift (Ratnakumar and Tunnacliffe 2006). These authors searched the deletion library of nonessential genes for those that affected desiccation tolerance specifically during the postdiauxic phase of growth. One quarter of the genes we identified also were identified by Ratnakumar and Tunnacliffe (2006) as being required for resistance to desiccation (Table 1). These include two of the six transcription factors (*MIG1* and *SUB1*) that we identified. They also noted that autophagy plays an important role in desiccation tolerance. We find that four autophagy genes are defective in R3 cell formation (*ATG16*, *ATG17*, *ATG27*, and *BRE5*).

Hundreds of genes are required for cells to survive a 30° to 50° heat shock, and they differ significantly depending on whether cells are growing exponentially or are in the stationary phase (Jarolim *et al.* 2013). This is further evidence that log phase and stationary phase are fundamentally different physiological states. We see very little overlap between these heat shock−sensitive mutants and the Rtd− mutants. However, if we look at genes required for acquired thermotolerance, there is significant overlap. Cells can acquire thermotolerance by a previous exposure to a nonlethal heat shock. This pretreatment induces the conserved family of heat shock proteins required to disaggregate and refold proteins that are denatured by subsequent heat treatments. Twenty-one mutants have been identified that fail to acquire thermotolerance after heat treatment (Mir *et al.* 2009), and half of these are among the mutants we identified (*SSD1*, *PDE2*, *RVS161*, *SPT3*, *APQ12*, *MNN11*, *RPL8A*, *MRC1*, and *PUN1*). This significant overlap between the genes that prepare cells to survive heat shock and those that prepare cells to enter quiescence may explain why quiescent cells are so much more heat tolerant than their nonquiescent siblings (Li *et al.* 2013). It also suggests that acquired stress tolerance is a key step in the transition to quiescence and that there may be novel components of this pathway among the Rtd- mutants. Consistent with this, one quarter of the Rtd- genes also promote ethanol tolerance (Table 1), including *SSD1* and *PDE2* (Sass *et al.* 1986; Avrahami-Moyal *et al.* 2012).

**Figure 4:**
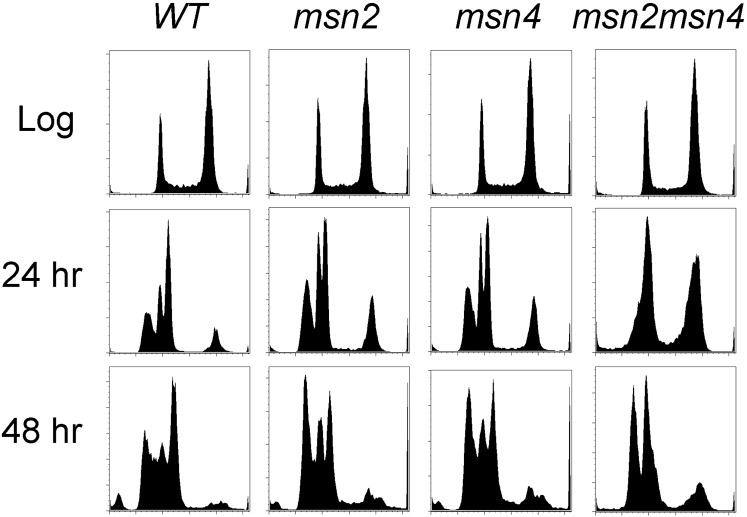
Msn2 and Msn4 contribute to the transition to quiescence. DNA fluorescence histograms of wild-type, *msn2*, *msn4*, and *msn2msn4* cells as indicated, during log phase, and after 24 and 48 hr of growth.

If acquired stress tolerance is a step in the transition to quiescence, we wondered whether Msn2 and/or Msn4 might play a role in this transition. Msn2 and Msn4 are structurally related master regulators of transcription in response to many stress conditions (Martinez-Pastor *et al.* 1996; Schmitt and McEntee 1996). They also induce transcripts at the diauxic shift that are normally repressed in high glucose by protein kinase A (PKA) (Boy-Marcotte *et al.* 1998). To see whether they are important for the transition to quiescence, we deleted one or both of these factors from a prototrophic W303 strain and assayed DNA fluorescence. Loss of one or both Msn proteins has no effect on the cytometry profile of log phase cells. After 24 hr of growth, the single mutants resemble wild type, but the double mutant shows no R3 peak and excess G2 cells (Figure 4). This finding suggests a redundant role for these stress response regulators in the transition to quiescence. Consistent with this we note that higher basal PKA activity resulting from a Pde2 mutation (Vandamme *et al.* 2012) has a similar phenotype (Figure 3). PKA-dependent phosphorylation of Msn2 prevents its nuclear import (Gorner *et al.* 1998). However, after 48 hr, we see a more wild-type pattern in the *msn2msn4* mutant (Figure 4), which indicates that the absence of Msn2 and Msn4 function delays, rather than prevents the production of R3 cells and the G1 arrest that is normally associated with quiescence.

### Quiescence may be a defense against the host’s immune response

Perhaps one of the most medically relevant aspects of the fungal cell wall is its impact on the fungal pathogens’ ability to evade their host’s immune response. Among the transcription factors with reduced R3 cells, Rim101 is the most striking because it and seven other components of the Rim101 activation pathway were picked up in our screen. Rim101 is implicated in cell wall assembly because of its sensitivity to cell wall stressors (Castrejon *et al.* 2006). This is a highly conserved pathway of cell wall regulation that also impacts fungal adaptation to its host (Lis *et al.* 2013; O’Meara *et al.* 2013, 2014).

Another well-studied example is Ssd1. Ssd1 is a conserved fungal protein that is polymorphic. It is known to affect the cell wall composition and virulence of *S. cerevisiae* in mice (Wheeler *et al.* 2003). It was also identified for its ability to confer resistance to antimicrobial peptides produced by plants (Ibeas *et al.* 2001; Tanaka *et al.* 2007) and animals (Gank *et al.* 2008). Ssd1 is an RNA binding protein that binds *ECM33* and a number of other mRNAs whose products are found in the cell wall and bud tip (Hogan *et al.* 2008; Jansen *et al.* 2009). *NAB6*, another Rtd− mutant, binds an overlapping set of mRNAs (Hogan *et al.* 2008). A genome-wide survey of the *S. cerevisiae* deletion library for mutants that reduce fitness in response to antimicrobial peptides identified Ssd1 and 22 other genes on our list (Lis *et al.* 2013). We have also shown that restoring the full-length Ssd1 protein to the W303 lab strain almost doubles Q-cell production and increases their thermotolerance and longevity (Li *et al.* 2009). These 23 mutants may identify components of the cell wall assembly pathway that are important for eluding the host defense mechanisms, and as such might be good targets for antifungal drugs. Another possibility is that cells that are most successful at entering quiescence are also most successful at evading their host’s immune response. If that is the case, other non-cell wall genes that promote quiescence may be a novel source of antifungal drug targets.

### Other genes implicated in regulation of quiescence

There are also R3-deficient mutants that are localized to sites of polarized cell growth (*P* = 0.14) (Bi and Park 2012). These include genes known to play roles in bud site selection (*BUD4*, *BUD14*, *BUD19*, and *BUD25*) and cytokinesis (*BNI5* and *CYK3*). These mutants could be compromising the cell wall, but they could also be regulating the asymmetrical cell division that cells transitioning to quiescence undergo, or controlling the rate of cell enlargement, which is significantly reduced during the transition to quiescence (Li *et al.* 2013).

We identified seven genes that are involved in translation. These could have a multitude of indirect effects on the transition to quiescence. However, one gene product of recent interest is *PUS1*. This gene encodes an enzyme that modifies uridine residues in rRNA, tRNA and other small nuclear RNAs to pseudouridine (ψ) and stabilizes them. Recently, ψ modifications were identified at hundreds of sites in mRNAs and noncoding RNAs in *S. cerevisiae* (Carlile *et al.* 2014). Moreover, the bulk of these targeted modifications are specific to the postdiauxic phase of growth. There are nine pseudouridine synthase (*PUS*), but Pus1 is responsible for the largest share of ψs in mRNAs. Subsequent studies in human HeLa cells showed a similar pseudouridylation of mRNAs, some of which were detected only after serum deprivation (Carlile *et al.* 2014). Hence, this regulated ψ modification of mRNAs is conserved from yeast to humans. It is known to stabilize RNA structure. It also could influence mRNA localization and translation efficiency. *In vitro*, it’s been shown that pseudouridylation of mRNAs facilitates noncanonical base pairing, which could selectively alter the genetic code in postdiauxic cells (Fernandez *et al.* 2013).

Protein degradation via the ubiquitin−proteosome system is also important, based on the need for three ubiquitin ligase components (*BUL1*, *MMS1*, and *HEL2*) and at least three proteasome components (*NAS2*, *VID28*, and *UMP1*). Interestingly, decreased proteasome function is a symptom of the aging process from yeast to humans (Chondrogianni *et al.* 2014) and proteasome function has previously been shown to be required for survival of stationary phase (Chen *et al.* 2004). Moreover, the overproduction of Ump1 promotes longevity (Chen *et al.* 2006). This raises the possibility that the life span promoting function of the ubiquitin-proteosome system is in part due to its role in transitioning cells to the quiescent state.

Mot3 is a transcription factor that activates several cell wall genes, including *CWP2 and SED1* (Holmes *et al.* 2013). Mot3’s role in expression of these genes could be responsible for the reduced R3 cell phenotype we observe in *mot3* mutants. However, Mot3 also inhibits genes in the ergosterol biosynthesis pathway, and it does so in cooperation with Sok2, another transcription factor we picked up in this screen (Chang *et al.* 2013). Ergosterol is the fungal equivalent of cholesterol and it is the main target of antifungal drugs. Ergosterol is produced by yeast cells when oxygen is available. Under hypoxic conditions, Mot3 represses *ERG* genes. Mot3 also forms self-propagating, detergent-resistant aggregates called prions. When aggregated in these prion forms, Mot3 is sequestered and incapable of regulating known target promoters (Alberti *et al.* 2009). The prion form of Mot3 is specifically induced by ethanol, and can be reversed by removal of oxygen (Holmes *et al.* 2013). These two signals mark the beginning and the end of the postdiauxic period of growth. Yeast cells ferment glucose to ethanol, then, when the glucose is gone (diauxie), they switch to respiration, which depletes oxygen from the local environment. These signals could evoke regulated prion switching of Mot3 during the growth cycle and influence gene expression in a rapid but reversible manner (Holmes *et al.* 2013).

Sub1 is a nonspecific DNA binding protein that has been implicated in transcription elongation, mRNA processing and DNA repair (Conesa and Acker 2010), but recent work indicates that it is also bound to promoters. Sub1 and its human paralog PC4 contain single-stranded DNA-binding domains, and evidence is accumulating that they bind single-stranded DNA and stabilize the open complex during initiation and elongation of transcription (Sikorski *et al.* 2011). Sub1 is also required for survival in unbuffered minimal media, but not rich media (Acker *et al.* 2014), and for desiccation tolerance (Ratnakumar and Tunnacliffe 2006). These phenotypes suggest an unanticipated role for Sub1 in the stress response and quiescence.

Another large class of Rtd− mutants play roles in DNA repair, chromosome cohesion, and chromosome segregation. Three of these (*NDJ1*, *SPO11*, and *SPO22*) are thought to be meiosis specific. The role of these proteins in the transition to quiescence is completely unexplored. Two other genes are involved in the DNA damage and replication stress checkpoints (*KIN3* and *MRC1*). Most of these mutants show a significant population of cells with G2 DNA content, suggesting a failure to G1 arrest, which is another critical step in the transition to quiescence. We know that there is replication stress during the transition to quiescence (Miles *et al.* 2013), so mutants defective in DNA repair could evoke a checkpoint arrest in S or G2. Mutants lacking checkpoint function would lose viability. Both of these defects would be likely to prevent the normal transition to quiescence and be scored as R3-deficient and G1 arrest-deficient. As with any screen carried out at a single time point in the growth cycle, differences in growth rate and responses to changing growth conditions will influence the rate of progression to quiescence. Hence, very slow-growing cells may not reach the diauxic shift after 24 hr of growth and would be in all stages of the cell cycle. For these reasons, mutants that appear to have failed to arrest in G1 require further study before such a conclusion can be validated.

Genes involved in metabolism and metabolic signaling also were identified. Carbon metabolism (*RHR2*), its regulation (*PDE2*, *PCL7*, and *SSK2*), and glucose-responsive transcription factors (*SOK2* and *MIG1*) are implicated. Phospholipid (*KCS1*, *INP52*, and *EPT1*) and methyltransferase activities (*SAM1*, *ADO1*, *MUP1*, *HPM1*, and *SEE1*) are also important for the transition to quiescence. Using YeastMine (Balakrishnan *et al.* 2012), we found that 18 of our mutants interact with the Set3 deacetylase complex (Table 1). This complex is important for modulating transcription in response to carbon source changes (Kim *et al.* 2012).

In summary, this screen serves as a starting point for the genetic dissection of the transition to quiescence in budding yeast. It was made possible by the fact that quiescent yeast cells can be distinguished by flow cytometry from their nonquiescent siblings. Stationary phase cultures of budding yeast are mixtures of four distinct cell types, which we refer to as R1 to R4. Only one of these cell types (R3) has achieved a protective, quiescent state, despite the fact that none of the cells are dividing. We have used flow cytometry to screen for deletions of nonessential genes that prevent the production of R3 cells. Many of these genes are implicated in the biogenesis of the cell wall, which is a uniquely elaborate structure in quiescent cells. The identification of these genes will help define the pathway and the components involved in Q-cell wall formation. These cell walls enable yeast cells to resist environmental stress and evade host immune responses. As such they are good targets for new antifungal drugs. Our results also implicate many other pathways in the transition to quiescence. Multiple genes involved in autophagy, ubiquitin-mediated proteolysis, DNA replication and repair, bud site selection, and cytokinesis were identified. In these instances, only a subset of the genes involved in these pathways were found in our screen. Our expectation is that these genes may lead us to a better understanding of how these major pathways respond to and tailor their activities to promote this major transition to a protective quiescent state.

## Supplementary Material

Supporting Information
