# Supplementary material for: A Genetic Screen for Saccharomyces cerevisiae Mutants That Fail to Enter Quiescence
Source: G3 (Bethesda). 2015 Jun 10;5(8):1783–95. doi: 10.1534/g3.115.019091 (PMC4528334; doi:10.1534/g3.115.019091)
Supplement: Supporting Information [file supp_5_8_1783__index.html]

A Genetic Screen for Saccharomyces cerevisiae Mutants that Fail to Enter Quiescence — A Genetic Screen for Saccharomyces cerevisiae Mutants That Fail to Enter Quiescence — Supporting Information 

# A Genetic Screen for *Saccharomyces cerevisiae* Mutants That Fail to Enter Quiescence

## Supporting Information for Li, Miles, and Breeden, 2015

**Files in this Data Supplement:**

- Supporting Information - Figures S1-S2 (PDF, 1 MB)
- Figure S1 - As budding yeast cultures grow from log phase to quiescence they differentiate into four distinct cell types. (PDF, 913 KB)
- Figure S2 - DNA peak with reduced fluorescence is unaltered by various incubation conditions. (PDF, 440 KB)
