# Supplementary material for: A Genetic Screen for Saccharomyces cerevisiae Mutants That Fail to Enter Quiescence
Source: G3 (Bethesda). 2015 Jun 10;5(8):1783–95. doi: 10.1534/g3.115.019091 (PMC4528334; doi:10.1534/g3.115.019091)
Supplement: Supporting Information [file supp_g3.115.019091_019091SI.pdf]

G3 · Genes | Genomes | Genetics

A genetic screen for *Saccharomyces cerevisiae* mutants that fail to enter quiescence.

Lihong Li*, Shawna Miles* and Linda L. Breeden*

*Basic Sciences Division

Fred Hutchinson Cancer Research Center

Seattle, Wa 98109

DOI: 10.1534/g3.115.019091

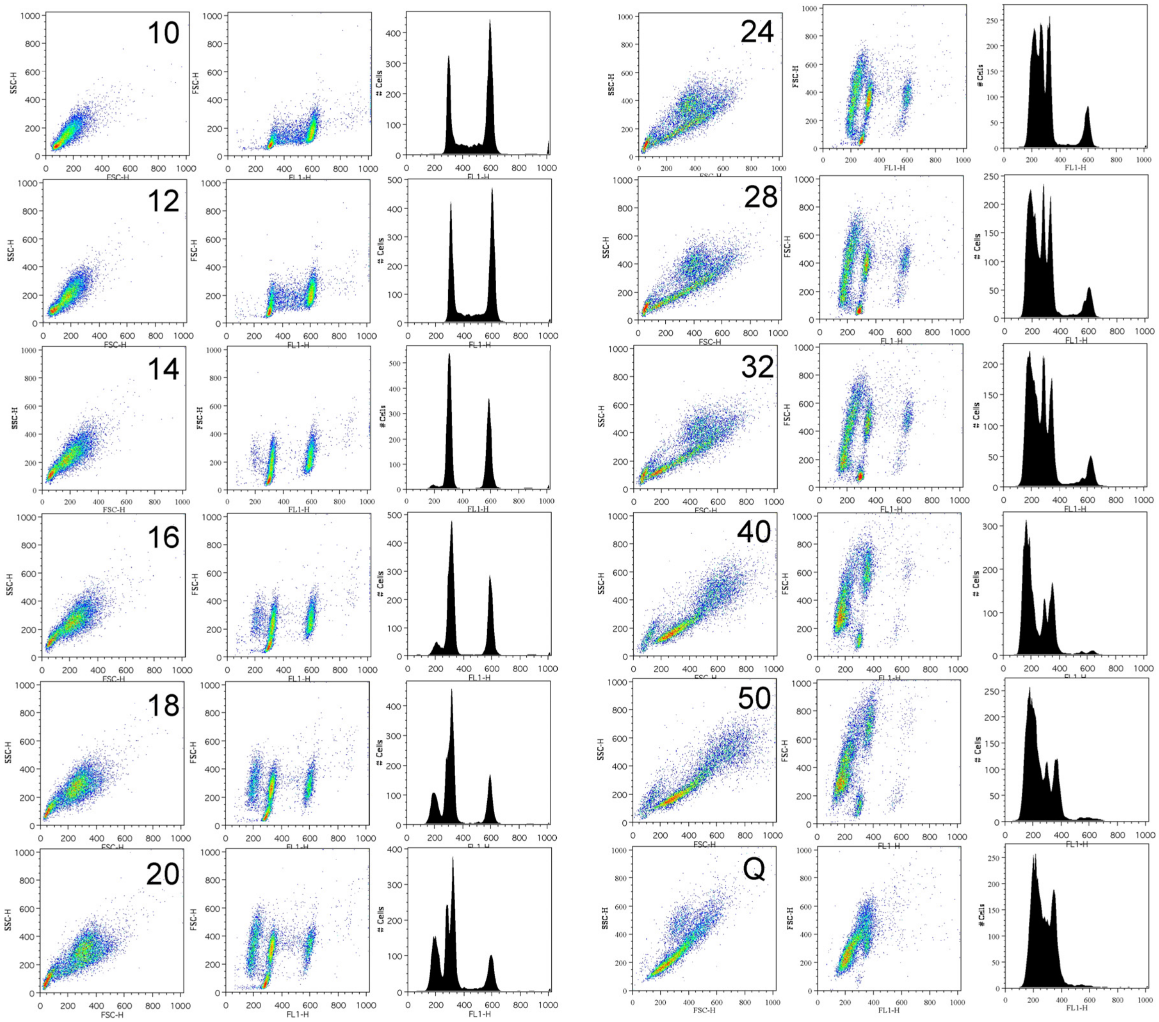

Figure S1 As budding yeast cultures grow from log phase to quiescence they differentiate into four distinct cell types. Using combinations of forward and side light scattering (FSC-H and SSC-H) and DNA fluorescence (FLH-1) we observe the appearance and disappearance of four cell types. Growth time in hours shown in upper left of left panels. Q indicates the cell type distribution in purified quiescent (Q) cells.

Li et al, Fig S2

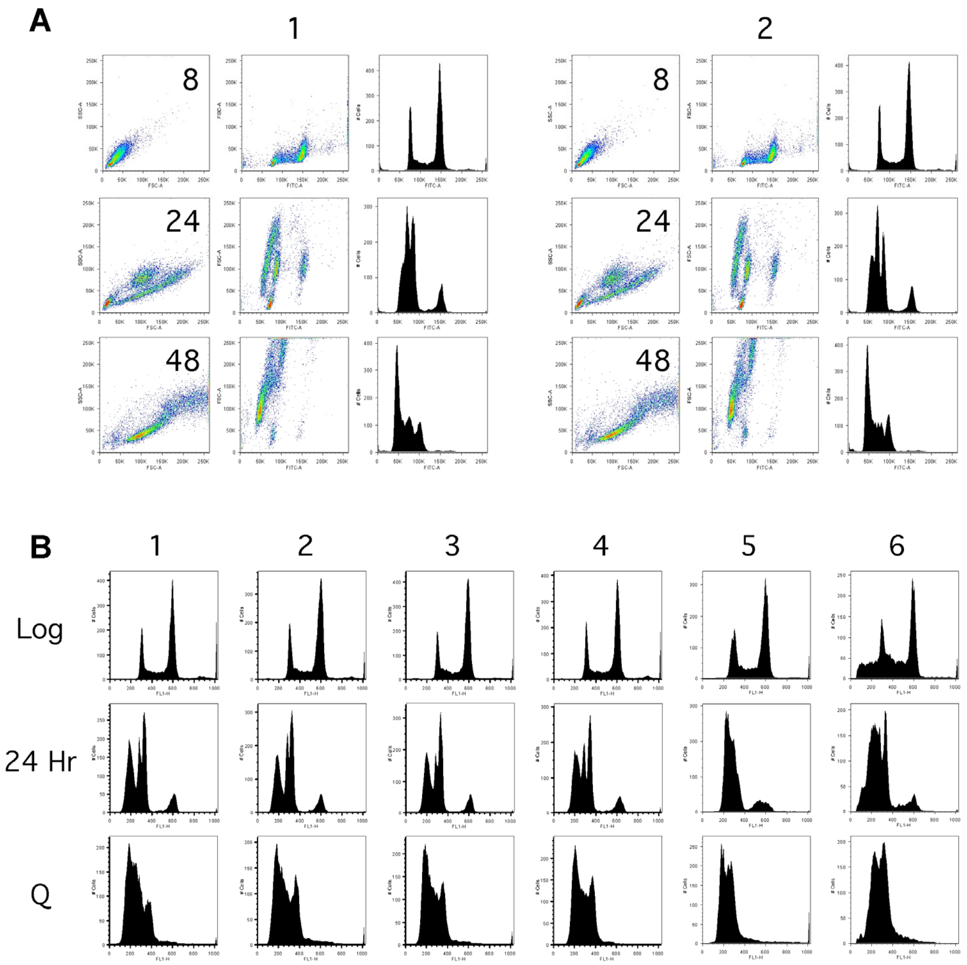

Figure S2 DNA peak with reduced fluorescence is unaltered by various incubation conditions. (A) A combination of forward and side light scattering (FSC-H and SSC-H) and DNA fluorescence (FLH-1) enables the tracking and quantification the peak with reduced fluorescence. One 24 hour old culture was prepared for flow cytometry by our standard protocol (see Materials and Methods) and then assayed one minute after Sytox Green dye addition (panel 1) and then again four days later (panel 2). We see no difference in the fluorescence intensity. (B) DNA fluorescence histograms of wild type cells in log phase, after 24 hours of growth and after Q cell purification were prepared for flow cytometry six different ways. 1) After our standard proteinase K digestion, cells were resuspended in .2M tris/HCl pH7.5, .2M NaCl, 78mM $MgCl_2$ and sonicated. Cells were pelleted, then resuspended in same plus Sytox Green, then diluted into Sytox Green in 50mM tris/HCl pH 7.5 one minute before analyzing. 2) Two hour RNAse treatment followed by one hour Proteinase K treatment, then both digestions were repeated before resuspending in

high salt and diluting into 50mM tris with dye as in 1) for one minute before analyzing. 3) Same as 2) except incubated with dye for four hours before analyzing. 4) Standard lab protocol (see Materials and Methods). 5) Haase and Reed protocol using RNAseA and pepsin digestions (Haase and Reed 2002). 6) Standard lab protocol with TritonX100 added to .25% to the final tris-dye mixture.
