# Supplementary material for: A Genetic Screen for Saccharomyces cerevisiae Mutants That Fail to Enter Quiescence
Source: G3 (Bethesda). 2015 Jun 10;5(8):1783–95. doi: 10.1534/g3.115.019091 (PMC4528334; doi:10.1534/g3.115.019091)
Supplement: Supporting Information [file supp_g3.115.019091_FigureS1.pdf]

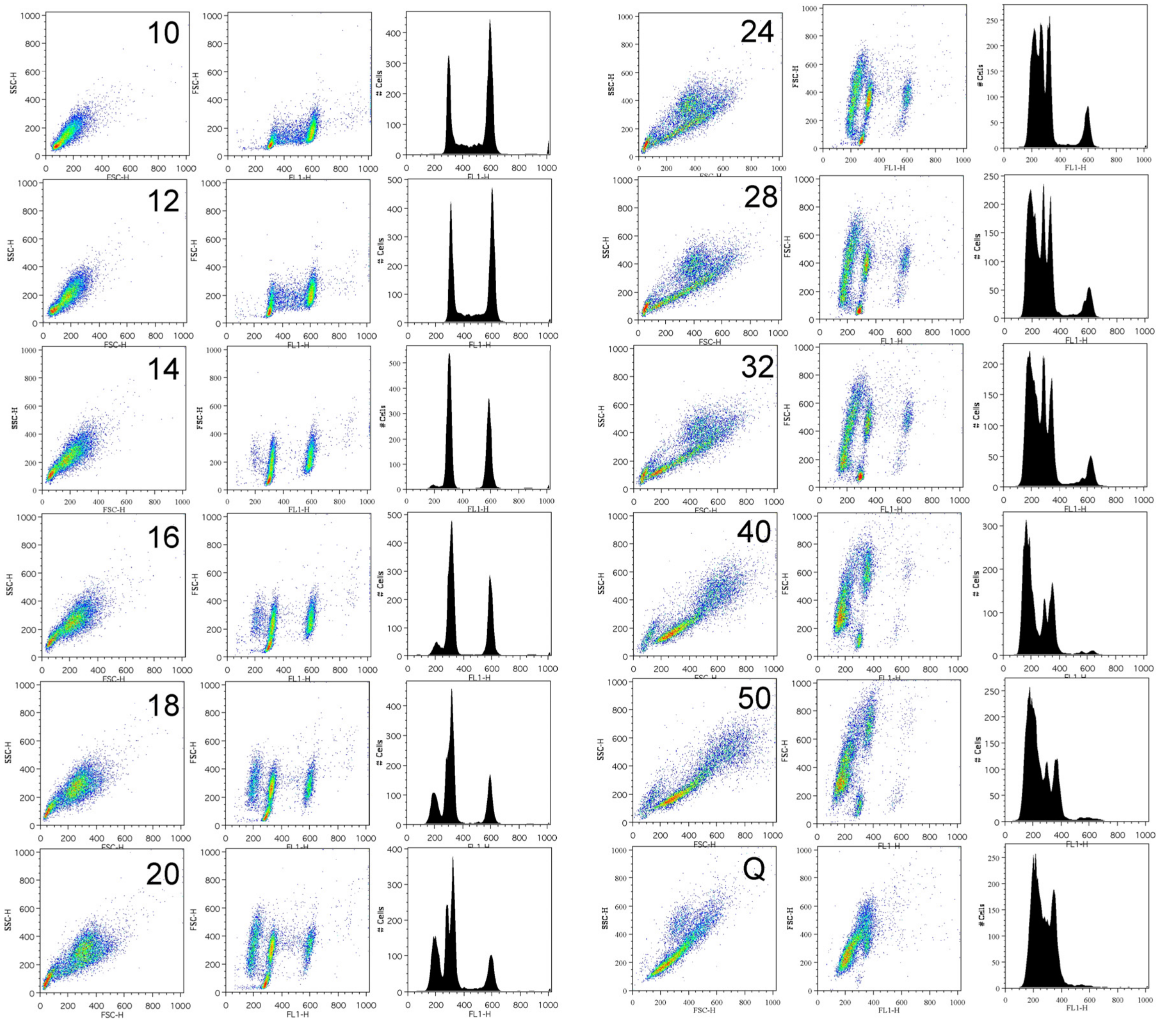

Figure S1 As budding yeast cultures grow from log phase to quiescence they differentiate into four distinct cell types. Using combinations of forward and side light scattering (FSC-H and SSC-H) and DNA fluorescence (FLH-1) we observe the appearance and disappearance of four cell types. Growth time in hours shown in upper left of left panels. Q indicates the cell type distribution in purified quiescent (Q) cells.
